# Comparison of Spinal Accessory Nerve Transfer versus C5 Grafting for Suprascapular Nerve Reinnervation in Brachial Plexus Birth Injury

**DOI:** 10.1097/PRS.0000000000012602

**Published:** 2025-11-11

**Authors:** Maria Hyttinen, Henrikki Rönkkö, Pasi Paavilainen, Jarkko Jokihaara

**Affiliations:** Tampere, Finland; From the 1 Faculty of Medicine and Health Technology, Tampere University; 2 Division of Musculoskeletal Diseases, Tampere University Hospital; 3 Pihlajalinna Hospital.

## Abstract

**Background:**

Nerve roots of the upper trunk are usually affected in brachial plexus birth injury. The suprascapular nerve originates from the upper trunk and innervates the infraspinatus muscle, which is the most important external rotator of the shoulder. Two common options for reinnervating the suprascapular nerve are from C5 with an interposition nerve graft or directly from the spinal accessory nerve. The aim of this study was to compare improvement in shoulder external rotation after brachial plexus reconstruction in patients with suprascapular nerve grafted from C5 or with spinal accessory nerve transfer.

**Methods:**

The authors retrospectively evaluated improvement of shoulder function in 16 patients after brachial plexus grafting with suprascapular nerve grafted from C5 and in 15 patients with concurrent spinal accessory nerve to suprascapular nerve transfer. In addition, results of secondary suprascapular nerve reinnervations with the spinal accessory nerve after failed nerve grafting (*n* = 7) were compared with the procedure performed at the primary surgery.

**Results:**

Mean improvement in external rotation was better in the nerve transfer group (mean 94 degrees [SD 61]) compared with the nerve graft group (mean 44 degrees [SD 54]; *P* = 0.023). The nerve graft group had an increased risk of secondary operations to improve active external rotation (*P* = 0.002), but not for anterior release procedures (*P* = 0.419). The outcome of spinal accessory nerve transfer was not diminished after primary grafting of the suprascapular nerve from C5 (mean 90 degrees [SD 37]; *P* = 0.875).

**Conclusion:**

Spinal accessory nerve transfer results in better active shoulder external rotation when compared with grafting of the suprascapular nerve from C5 and reduces the risk of secondary surgery.

Brachial plexus birth injury (BPBI) results from traction during delivery and causes temporary or permanent deficiency in upper extremity functions. The incidence of BPBI has been reported to be 0.9 to 5.1 per 1000 live births.^1–3^ Nerve roots of the upper trunk (C5 and C6) are usually affected,^4^ and the most popular surgical treatment is neuroma resection and brachial plexus reconstruction with sural grafts.^5–7^ Distal nerve transfers, such as spinal accessory nerve to suprascapular nerve transfers (SAN–SSN) for shoulder external rotation (ER), have been increasingly used. SAN–SSN has traditionally been used in cases of avulsions of upper trunk nerve roots or if other functions have recovered but deficiency remains in shoulder ER.^6^ Providing an additional source for regenerating motor axons, nerve transfers—especially the dorsal approach for SAN–SSN—offer a shorter regenerating distance for axons^8,9^ and surgical dissection outside the injured and scarred area without the need for nerve grafts.^9^ The use of SAN–SSN concurrently with reconstruction of the upper trunk without grafting from the C5 root to the SSN branch has been advocated,^10–13^ but evidence of benefits is unclear.

We compared recovery of ER after brachial plexus reconstruction with either C5–SSN or SAN–SSN and evaluated whether the results of secondary SAN–SSN operations after primary graft reconstruction are worse due to time delay when compared with primary SAN–SSN operations.

## PATIENTS AND METHODS

**Fig. 1.**
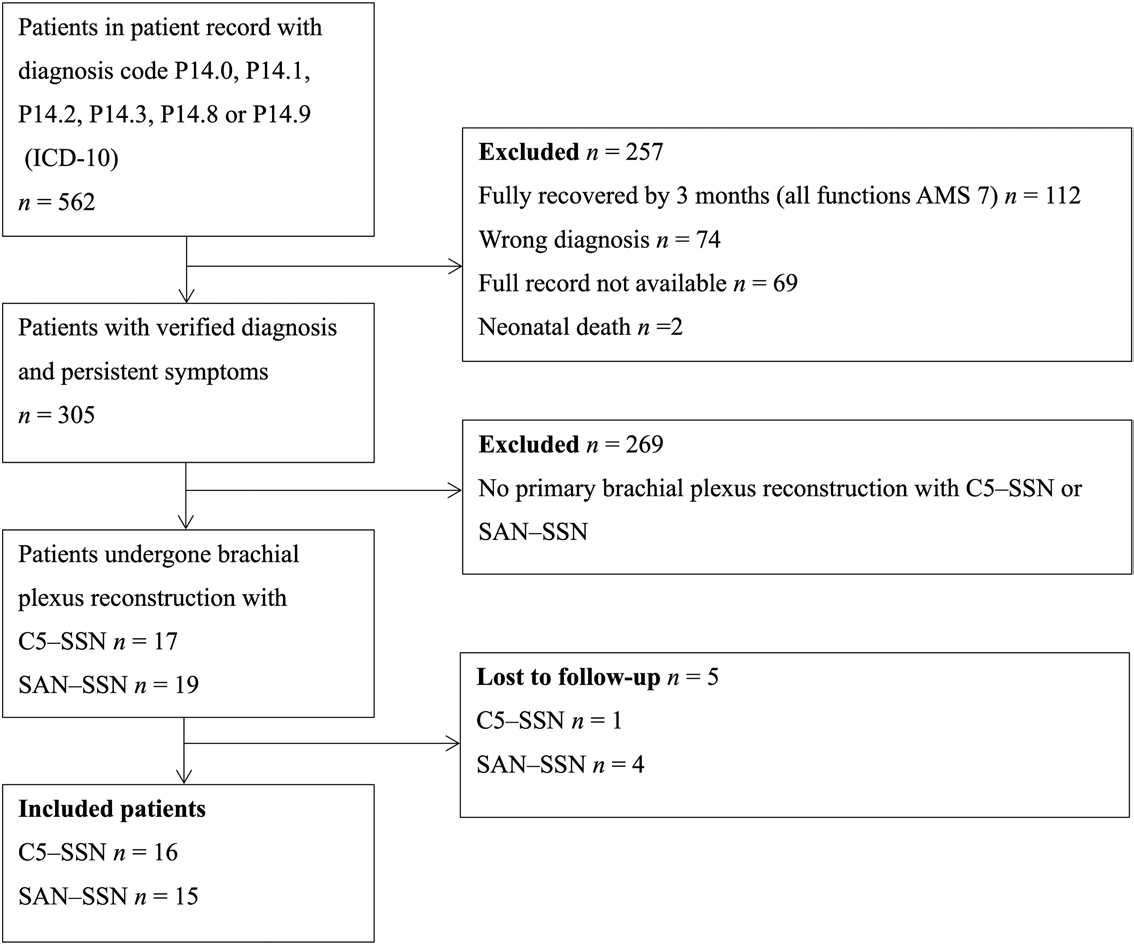
Study flowchart.

We screened patients born between 1999 and 2021 with diagnosis codes for birth injuries to the peripheral nervous system (International Classification of Diseases, 10th revision codes P14.0, P14.1, P14.2, P14.3, P14.8, or P14.9) treated at the authors’ institutions. Patients with an incorrect diagnosis, full spontaneous recovery before 3 months, or incomplete records were excluded. Of the 305 patients with confirmed BPBI, we included those who had undergone a brachial plexus reconstruction with C5–SSN or SAN–SSN. Patients with no data on preoperative assessment or with less than 24 months of postoperative follow-up were excluded (Fig. 1).

Patients referred to early operative treatment had persistent and severe symptoms at the age of 3 to 4 months. Magnetic resonance imaging was conducted to detect possible root avulsions. Absence of antigravity elbow flexion after 3 months was regarded as an indication for surgical treatment.^5,14,15^ The postoperative treatment protocol consists of systematic assessments every 3 to 6 months, starting from 2 months postoperatively, to 2 years. After that, evaluations are performed 1 or 2 times a year. Clinical evaluations were performed independently by a hand surgeon specialized in BPBI and a physiotherapist. The same surgeons operated on the patients and performed the clinical evaluations. Active and passive ER were assessed in shoulder adduction with the elbow at 90 degrees, starting with internal rotation with the palm touching the abdomen. The arm in the sagittal plane in front of the body was regarded as the neutral position (0 degrees). The maximum range of movement was from −90 to 90 degrees (ie, 180 degrees).

Passive ER was observed during the follow-up, and if any sign of internal rotation contracture was detected before 1.5 years of age, the patient was treated with botulinum toxin type A (BoNT-A) injections to the shoulder internal rotator muscles and a spica brace in external rotation for 6 weeks. This protocol was not used in patients treated in the beginning of the study period, as BoNT-A injections for this indication were not available in our hospital until 2006.

Details of the operations, injury type based on intraoperative assessment, and preoperative and postoperative ranges of motion in ER were collected from the electronic patient records. The primary outcome was improvement in ER between the last preoperative and the final postoperative clinical evaluation, measured as a continuous variable in degrees. Active ER at the final evaluation was assessed using the Active Movement Scale (AMS)^16^ (from 0 to 7 [best], in which 5 is motion less than or equal to one-half range and 6 is motion greater than one-half range against gravity, and AMS grade 6 [ie, neutral position] is considered sufficient function); AMS grade 6 (ie, neutral position) was considered sufficient function. If the patient was referred to secondary surgery (eg, SAN–SSN, tendon transfer) for ER during the postoperative follow-up, time of the secondary operation was regarded as the end of follow-up for the primary operation.

### Surgical Procedures

Brachial plexus exploration and reconstruction were performed through a supraclavicular incision. If there were no preganglionic avulsion injuries in the upper trunk, and if proximal stumps of C5 or C6 were viable based on visual inspection, intraoperative nerve stimulation, and frozen tissue samples, reconstruction of anterior and posterior divisions was conducted. Neuromas were resected and the nerve roots were reconstructed with interposition nerve grafts using sural nerves as donor nerves, with additional grafts from antebrachial cutaneous nerves if needed. A total of 3 grafts was typical: 2 to the upper trunk and 1 to the SSN.

SSN, which originates from the upper trunk at the Erb point and innervates the supraspinatus and infraspinatus muscles,^17^ was either reconstructed with a graft from C5 or neurotized with nerve transfer from SAN concurrently with grafting of anterior and posterior divisions. In SAN–SSN procedures, SAN was cut as distally as possible and directly coaptated end to end with SSN.

In our cohort, SAN–SSN procedures were performed through an anterior approach, through the same incision with concurrent grafting. All secondary SAN–SSN procedures were performed using a dorsal approach through an incision along the spine of the scapula. The SAN was coaptated to either the main trunk of the suprascapular nerve or a branch of the suprascapular nerve leading to the infraspinatus muscle (SAN–SSN–IB).^18^ The decision about secondary SAN–SSN was made based on incomplete motor recovery of ER. Latissimus dorsi and teres major (LD/TM) to infraspinatus tendon transfer was used in secondary operations before SAN–SSN became the standard practice. Tendon transfers are used at a later stage in patients with incomplete recovery of ER after primary SAN–SSN.

### Statistical Methods

Depending on the distribution, results are presented as mean ± SD or median (interquartile range [IQR]). R version 4.3.2 (R Foundation for Statistical Computing) was used for statistical analyses. Postoperative improvement in ER was tested with a Wilcoxon signed rank test. An independent samples *t* test or a Mann-Whitney *U* test was conducted to compare the means or medians between the groups, and a Fisher exact test to compare categorical variables. Cox proportional hazards (PH) regression, with assumptions confirmed with the Schoenfeld residuals test, was conducted to study the recovery of ER to AMS 6 after primary surgery and the risk for needing secondary surgery for ER. Kaplan-Meier survival plots are presented regarding the risk for secondary operations for active ER and anterior release. The level of statistical significance was set to *P* < 0.05.

## RESULTS

Sixteen patients who had undergone brachial plexus reconstruction with C5–SSN and 15 who had undergone brachial plexus reconstruction with SAN–SSN were included in our study (Table 1). The mean age at operation was 5 months (SD 1) for C5–SSN and 3 months (SD 1) for SAN–SSN (*t* test; *P* = 0.022). All the patients had preoperative active ER of −90 degrees (no active ER; AMS 0) and free passive ER and congruent shoulder joint at the time of the primary surgery. Presence of avulsion (Fisher exact test; *P* = 0.252) and involvement of lower roots C8 and TH1 (Fisher exact test; *P* = 0.149) did not differ across the primary surgery groups. The duration of postoperative follow-up until the last available patient record or secondary surgery for active ER was a median of 4.9 years (IQR 13.2) for the C5–SSN group and 11.0 years (IQR 8.2) for the SAN–SSN group.

**Table 1.** Patient Characteristics, ER after Primary Surgery, and Details of Secondary Surgery in Patients with Brachial Plexus Reconstruction with C5–SSN or SAN–SSN^a^

Patient Characteristics and Surgical Details	C5–SSN (*n* = 16)	SAN–SSN (*n* = 15)	*P*
Sex			0.473
Male	5	7	
Female	11	8	
Level of injury			0.352
C5–C6	6	3	
C5–C6–C7	6	4	
C5–C6–C7–C8	3	4	
C5–C6–C7–C8–TH1	1	4	
Age at surgery, mo	5 ± 1	3 ± 1	0.022^b^
Postoperative follow-up, yrs	4.9 (13.2)	11.0 (8.2)	0.199
Improvement in ER, degrees	44 ± 5	60 ± 61	0.023^b^
Final outcome in ER, degrees	−46 ± 53	4 ± 61	0.023^b^
ER at the final evaluation, AMS grade			0.103
0–4	9	4	
5	1	—	
6	6	11	
7	—	—	
Secondary surgery			
Tendon transfer for ER without anterior release	1	1	
Tendon transfer for ER with anterior release	2	1	
Anterior release	—	4	
Secondary SAN–SSN without anterior release	5	—	
Secondary SAN–SSN with anterior release	3	—	

a Data are presented as *n*, mean ± SD, or median (interquartile range).

b Significant.

### Results of Primary Operations

**Fig. 2.**
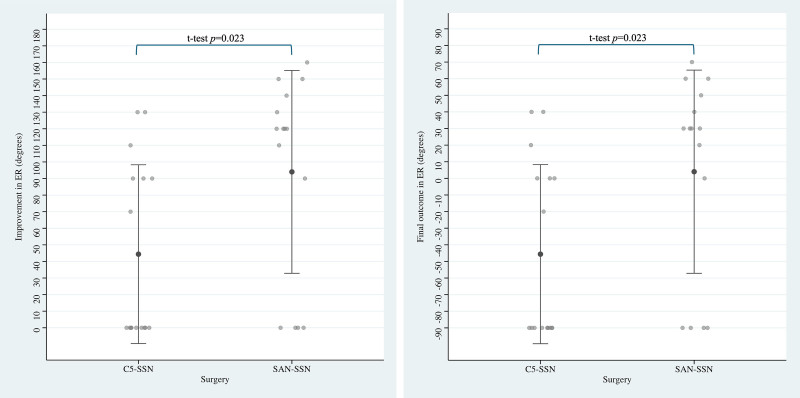
(*Left*) Improvement and (*right*) final outcome in active shoulder ER range of motion in degrees after brachial plexus reconstruction surgery with C5–SSN or SAN–SSN. *Gray dots* represent individual patients, *black dot* represents the mean, and *whiskers* represent the SD.

ER improved a mean of 44 degrees (SD 54) (Wilcoxon test; *P* = 0.017) after brachial plexus reconstruction with C5–SSN and 94 degrees (SD 61) (Wilcoxon test; *P* = 0.003) after brachial plexus reconstruction with SAN–SSN (Fig. 2, *left*). After C5–SSN, 6 of 16 patients achieved ER AMS 6; after SAN–SSN, 11 of 15 patients achieved ER AMS 6. Comparison between primary brachial plexus reconstruction with C5–SSN or SAN–SSN revealed a significant difference in mean ER improvement between the primary operations (*t* test; *P* = 0.023) and in the final range of ER (C5–SSN, mean −46 ± 53 degrees; SAN–SSN, mean 4 ± 61 degrees; *t* test; *P* = 0.023) (Fig. 2, *right*). Time from surgery to recovery of ER to AMS 6 was a mean of 20 ± 7 months after C5–SSN and 42 ± 17 months after SAN–SSN (*t* test; *P* = 0.002).

In a Cox PH model adjusted for age at surgery, passive restriction of ER less than 70 degrees during the follow-up, avulsion or involvement of lower roots, and primary surgery method were not associated with recovery of ER to AMS 6 (*P* = 0.283).

### Secondary Surgery for ER

Eleven of 16 patients in the C5–SSN group required secondary surgery due to insufficient active ER. Eight of the patients underwent SAN–SSN, 1 of whom was lost to follow-up. Five of the operations were SAN–SSN–IB. Details about secondary procedures are presented in Tables 1 and 2. The mean improvement in ER after secondary SAN–SSN was 90 ± 37 degrees (Wilcoxon test; *P* = 0.018), with all the patients achieving AMS 6 ER (Table 2) by a mean of 11 ± 4 months. A significant difference was found in time to AMS 6 recovery in primary brachial plexus reconstruction with SAN–SSN versus secondary SAN–SSN (*t* test; *P* < 0.001), but not in median ER improvement (*t* test; *P* = 0.875) or final active ER (*t* test; *P* = 0.323).

**Table 2.** Injury Characteristics and Outcome of the Secondary SAN–SSN Operations (*n* = 7)

Level of Injury	Values^a^
C5–C6	1
C5–C6–C7	2
C5–C6–C7–C8	2
C5–C6–C7–C8–TH1	1
Age at surgery, mo	25 ± 7
Preoperative ER, degrees	−64 ± 44
Postoperative follow-up, yrs	3.6 (1.9)
Improvement in ER, degrees	90 ± 37
Final range in ER, degrees	29 ± 25
ER at the final evaluation, AMS grade	
0–4	—
5	—
6	7
7	—

a Data are presented as *n*, mean ± SD, or median (interquartile range).

After primary brachial plexus reconstruction with concurrent SAN–SSN, 2 patients needed a secondary surgery (LD/TM tendon to infraspinatus tendon transfer) with AMS 0 active ER at ages 24 and 42 months, 19 and 39 months from the primary surgery, respectively.

**Fig. 3.**
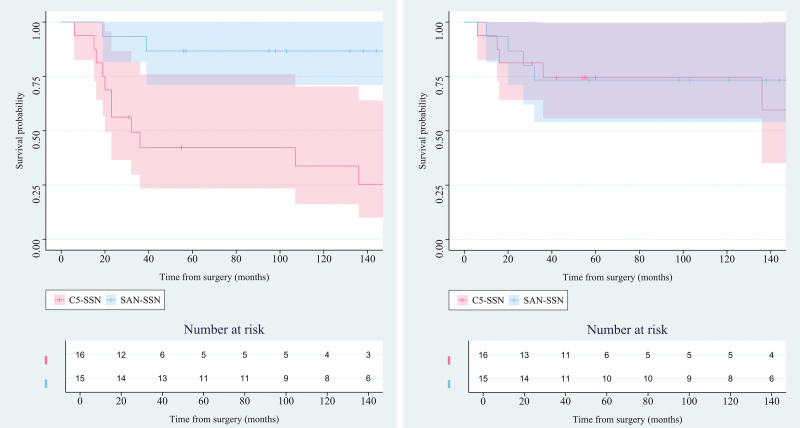
(*Left*) Survival probability without secondary surgery for shoulder ER after brachial plexus reconstruction surgery with C5–SSN or SAN–SSN, with 95% confidence intervals. Secondary procedures were SAN–SSN or a tendon transfer procedure for ER (LD/TM tendon to infraspinatus tendon transfer). Log-rank *P* = 0.002. (*Right*) Survival probability without anterior release of glenohumeral joint capsule and possible subscapular tendon lengthening after brachial plexus reconstruction surgery with C5–SSN or SAN–SSN, with 95% confidence intervals. Anterior release was performed if passive restriction of shoulder ER was detected after 1.5 years of age. Log-rank *P* = 0.700.

The risk of requiring a secondary procedure (SAN–SSN or LD/TM to infraspinatus tendon transfer) was higher in the C5–SSN group (log-rank *P* = 0.002) (Fig. 3, *left*). The median time from the primary to the secondary operation was 23 months (IQR, 20 months). In a Cox PH model adjusted for age at surgery, passive ER less than 70 degrees during follow-up, and avulsion or involvement of the lower roots, C5–SSN was associated with a greater risk for needing a secondary surgery to restore active ER (*P* = 0.013). Involvement of lower roots was also associated with increased risk (*P* = 0.043). (**See Table, Supplemental Digital Content 1**, which shows Cox PH regression for recovery of ER to AMS 6 after C5–SSN or SAN–SSN in primary brachial plexus reconstruction, https://links.lww.com/PRS/I560.)

### Passive ER

There was no difference between the surgery groups in whether the patients had been operated on before or after the beginning of BoNT-A injections (Fisher exact test; *P* = 1.000). A total of 21 patients had some level of restriction of passive ER (<70 degrees) (12 in the C5–SSN group and 9 in the SAN–SSN group) during the follow-up, and a total of 9 anterior capsule release and subscapular tendon lengthening procedures were performed in patients with passive ER restricted to neutral position (Table 1). Evolution of passive ER restriction less than 70 degrees during the follow-up was associated with lower probability of achieving ER AMS 6 in the adjusted Cox PH model (*P* = 0.015), but was consistent across the surgery groups (Fisher exact test; *P* = 0.458). (**See Table, Supplemental Digital Content 2**, which shows Cox PH regression for secondary surgery for active ER [SAN–SSN or LD/TM to infraspinatus tendon transfer] after SAN–SSN or C5–SSN in primary brachial plexus reconstruction, https://links.lww.com/PRS/I561.) There was no difference in the risk of anterior release surgery between the groups (log-rank *P* = 0.700) (Fig. 3, *right*).

## DISCUSSION

We evaluated the active ER outcome in patients with BPBI who underwent C5–SSN or SAN–SSN with concomitant brachial plexus reconstruction. Improvement in ER after C5–SSN was poorer compared with SAN–SSN procedures. Nevertheless, there was no difference in improvement of ER after SAN–SSN operations performed at a later age after failed C5–SSN when compared with primary SAN–SSN performed concurrently with brachial plexus reconstruction. Patients treated with C5–SSN more often needed secondary surgery for improving poor active ER. SAN–SSN is an effective treatment option for reinnervating SSN in primary surgery and is also usable in patients with poor ER when grafting is not feasible.

Our findings on outcome in shoulder function and risks for secondary surgery are in agreement with previous reports comparing the procedures.^10,11,13^ Segal et al.^12^ reported good improvement after SAN–SSN transfers performed concurrently with upper trunk reconstruction in 27 patients, but results were not compared with only nerve grafting. Nevertheless, in these studies, ER outcomes were evaluated using the AMS. As an ordinal scale, AMS may not be optimal for statistical evaluation of improvement in ER; AMS 6 is a wide class, including patients with neutral movement to those with nearly full ER. In the current study, the longer time to AMS 6 recovery after SAN–SSN influenced the survival analysis, which may explain the lack of a detected difference in recovery to AMS 6 between groups, despite superior ER outcomes in the SAN–SSN group. Because recovery of function is gradual and recovery occurs between the follow-up evaluations, further conclusions from the longer time taken to AMS 6 recovery after primary SAN–SSN compared with C5–SSN cannot be drawn. Due to the difficulty of evaluating shoulder movements in children, the estimates of active range of motion in ER may be inaccurate; therefore, assessments by 2 independent professionals were used to reduce measurement error.

In contrast to other studies, in a study by Pondaag et al.,^19^ results of SAN–SSN and C5–SSN were both poor. Only 25 of 65 patients in the C5–SSN group and 6 of 11 in the SAN–SSN group achieved active ER AMS 6 when measuring the isolated glenohumeral movement. However, functional results in Mallet hand-to-mouth and hand-to-head grading^20^ were better than results in ER in both groups, which may result from compensatory movement. In our study, ER was measured in full adduction to isolate the glenohumeral movement, but measurement of joint movement is still prone to error.

Preceding failed suprascapular nerve grafting (C5–SSN) seemed not to worsen the results of secondary SAN–SSN. Nevertheless, the number of patients who underwent SAN–SSN as a secondary procedure was low in our cohort. Sommarhem et al.^18^ and Grahn et al.^21^ reported a mean ER improvement of 47 to 48 degrees in patients treated with SAN–SSN–IB at a mean age of 2 to 3 years, but all the patients in their series had at least AMS 5 preoperative ER. In our recent study, patients operated at a mean age of 22 months had a median improvement of ER of 110 degrees.^22^ The SAN–SSN outcome may be reduced if the operation is delayed,^22,23^ and the decision about surgery is suggested to be made by 1.5 years of age.^22^ According to our results, performing C5–SSN at the primary operation leads to uncertain recovery of ER and increased risk for secondary operations. However, the anterior approach to SAN–SSN seems to delay the recovery of ER. Prolonged absence of active ER causes imbalance in shoulder muscles and leads to passive restrictions of movement and glenohumeral joint incongruence,^24–26^ which further hinders the recovery of active movement. Thus, although results of secondary SAN–SSN operations were not significantly poorer when compared with results of SAN–SSN performed at the primary surgery, prolonged denervation of muscles may result in various sequelae.

Several factors may affect the outcome of nerve grafting or nerve transfer surgery. Intraoperative assessment of proximal nerve stumps is crucial in sural nerve grafting and must be confirmed with histopathologic analysis, because grafting a root with a low number of active neuronal units results in a poor outcome.^27^ Distal cut of a spinal accessory nerve may lead to poorer results, as the number of axons available is smaller than in the proximal part.^19^ Peripheral nerve transfers, such as SAN–SSN, have shown good results in multiple studies.^10,11,28^ However, appropriate patient selection for surgical treatment and appropriate age for the decision about a surgical procedure are well-known challenges when making treatment decisions for BPBI.

An important consideration in the care of patients with BPBI is hand sensibility, which can be significantly reduced and affects overall hand function. Reduced sensory function has been reported in conservatively and surgically treated patients, even with C5–C6 upper plexus injury.^29^ SAN–SSN procedures aim to restore motor function,^30^ and do not restore sensory function. More research is needed to discern the effect of grafting on sensory recovery in C5–C6 injury. If SAN–SSN is performed concurrently with brachial plexus reconstruction in the early phase, the advantages of SAN–SSN and grafting can be combined. Nevertheless, nerve transfers may sporadically result in donor-site morbidity, which manifests as dysfunction of the trapezius muscle when SAN is used as a donor nerve.^30^ This was not evaluated in this study.

Our study is limited by the retrospective study design, small cohort, and heterogeneity of the lesions. Evaluation of injury severity in small children with BPBI is difficult. The treatment protocol in our hospital changed during the study period with the introduction of BoNT-A injections in our hospital, and not all the possible factors can be considered when comparing the study groups. In addition, the primary SAN–SSN procedures in this study cohort were done with an anterior approach, although the current treatment protocol in our hospital favors a dorsal approach. In the literature, slightly better or similar outcomes in ER have been reported with the dorsal approach compared with the more traditional anterior approach.^31,32^ Furthermore, the regeneration distance of the nerve fibers to the motor endplate is longer, which may result in slower recovery of ER.^31,33^ In our study, recovery of ER occurred sooner after secondary dorsal SAN–SSN procedures versus primary anterior SAN–SSN procedures. The majority of secondary SAN–SSN operations were SAN–SSN–IB, which may allow for faster recovery of ER by directing axonal growth specifically to the infraspinatus muscle rather than also innervating the supraspinatus. However, simultaneous anterior release operations performed with secondary SAN–SSN may have influenced the results. Whether release of the internal rotation contracture alone would have been sufficient to enable already recovered motor function to produce active movement is unknown.

The use of nerve transfer surgery in pediatric patients has been increasing.^6^ Results of nerve transfers are favorable, and because the shorter distance to the target muscle allows for faster reinnervation, these procedures are also suitable for later operations.^34^ Regarding whether reconstruction of the upper trunk in BPBI is better performed using C5–SSN or a concurrent SAN–SSN transfer, our results suggest that concurrent SAN–SSN provides superior recovery of external rotation compared with C5–SSN. Concurrent SAN–SSN is an effective primary treatment option and enables saving grafts for reconstruction of the brachial plexus.

## DISCLOSURE

The authors declare no potential conflicts of interest with respect to the research, authorship, or publication of this article. No financial support was received for the research, authorship, or publication of this article.

## Supplementary Material


