# Supplementary material for: Comparison of Spinal Accessory Nerve Transfer versus C5 Grafting for Suprascapular Nerve Reinnervation in Brachial Plexus Birth Injury
Source: Plast Reconstr Surg. 2025 Nov 11;158(1):76–84. doi: 10.1097/PRS.0000000000012602 (PMC13290028; doi:10.1097/PRS.0000000000012602)
Supplement: Supplementary file 1 [file prs-158-076e-s001.pdf]

**Table, Supplemental Digital Content 1.** Cox proportional hazards regression for ending up in secondary surgery for active ER (SAN–SSN or LD/TM to infraspinatus tendon transfer) following SAN–SSN or C5–SSN in primary brachial plexus reconstruction.

| | | CHR | 95% CI | *p*-value | AHR | 95% CI | *p*-value |
|---|---|---|---|---|---|---|---|
| **Surgery** | | | | 0.010* | | | 0.013* |
| | **C5–SSN** | 1 | – | – | 1 | – | – |
| | **SAN–SSN** | 0.13 | 0.03–0.62 | 0.010* | 0.01 | <0.01–0.39 | 0.013* |
| **Age at surgery** | | 1.74 | 1.04–2.91 | 0.035* | 0.93 | 0.43–2.03 | 0.861 |
| **Presence of avulsion** | | | | 0.198 | | | 0.115 |
| | **No** | 1 | – | – | 1 | – | – |
| | **Yes** | 0.44 | 0.13–1.53 | 0.198 | 0.11 | 0.01–1.71 | 0.115 |
| **Involvement of C8 or TH1** | | | | 0.673 | | | 0.043* |
| | **No** | 1 | – | – | 1 | – | – |
| | **Yes** | 1.29 | 0.39–4.35 | 0.673 | 16.68 | 1.08–257.23 | 0.043* |
| **Passive restriction <70 degrees** | | | | 0.331 | | | 0.823 |
| | **No** | 1 | – | – | 1 | – | – |
| | **Yes** | 2.14 | 0.46–9.94 | 0.331 | 0.81 | 0.13–5.18 | 0.823 |

ER=shoulder external rotation; SAN–SSN=spinal accessory nerve to suprascapular nerve transfer; LD/TM=latissimus dorsi/teres major; C5–SSN=grafting of suprascapular nerve from C5 nerve root; CHR=crude hazard ratio; CI=confidence interval; AHR=adjusted hazard ratio
