# Supplementary material for: Comparison of Spinal Accessory Nerve Transfer versus C5 Grafting for Suprascapular Nerve Reinnervation in Brachial Plexus Birth Injury
Source: Plast Reconstr Surg. 2025 Nov 11;158(1):76–84. doi: 10.1097/PRS.0000000000012602 (PMC13290028; doi:10.1097/PRS.0000000000012602)
Supplement: Supplementary file 2 [file prs-158-076e-s002.pdf]

Table, Supplemental Digital Content 2. Cox proportional hazards regression for recovery of ER to AMS 6 level following C5–SSN or SAN–SSN in primary brachial plexus reconstruction.

		CHR	95% CI	*p*-value	AHR	95% CI	*p*-value
**Surgery**				0.985			0.283
	**C5–SSN**	1	–	–	1	–	–
	**SAN–SSN**	1.01	0.37–2.77	0.985	0.41	0.08–2.08	0.283
**Age at surgery**		1.08	0.73–1.60	0.711	1.41	0.80–2.50	0.236
**Passive restriction <70**				0.018*			0.015*
	**No**	1	–	–	1	–	–
	**Yes**	0.30	0.11–0.81	0.018*	0.16	0.04–0.70	0.015*
**Presence of avulsion**				0.282			0.491
	**No**	1	–	–	1	–	–
	**Yes**	0.55	0.19–1.63	0.282	0.56	0.10–2.96	0.491
**Involvement of C8 or TH1**				0.820			0.679
	**No**	1	–	–	1	–	–
	**Yes**	0.89	0.33–2.43	0.820	1.43	0.26–7.82	0.679

ER=shoulder external rotation; AMS=Active Movement Scale; C5–SSN=grafting of suprascapular nerve from C5 nerve root; SAN–SSN=spinal accessory nerve to suprascapular nerve transfer; CHR=crude hazard ratio; CI=confidence interval; AHR=adjusted hazard ratio
